# Was the Maternal Health Cash Transfer Programme in Nigeria Sustainable and Cost-Effective?

**DOI:** 10.3389/fpubh.2020.582072

**Published:** 2020-11-04

**Authors:** Obinna Onwujekwe, Tim Ensor, Pamela Ogbozor, Chinyere Okeke, Uche Ezenwaka, Joseph P. Hicks, Enyi Etiaba, Benjamin Uzochukwu, Bassey Ebenso, Tolib Mirzoev

**Affiliations:** ^1^Health Policy Research Group, Department of Pharmacology and Therapeutics, College of Medicine, University of Nigeria Enugu Campus, Nsukka, Nigeria; ^2^Department of Health Administration and Management, College of Medicine, University of Nigeria Enugu Campus, Nsukka, Nigeria; ^3^Nuffield Centre for International Health and Development, Leeds Institute of Health Sciences, University of Leeds, Leeds, United Kingdom; ^4^Department of Psychology, Enugu State University of Science and Technology, Enugu, Nigeria

**Keywords:** interrupted time series, Nigeria, cost effectiveness, maternal health, conditional cash transfer (CCT)

## Abstract

**Background:** The Subsidy Reinvestment and Empowerment Programme (SURE-P), Maternal and Child Health (MCH) was introduced by the Nigerian government to increase the use of skilled maternal health services and reduce maternal mortality. The programme, funded out of a reduction in the fuel subsidy, was implemented between October 2012 and April 2015 and incorporated a conditional cash transfer to women to encourage use of facility based maternal services. We seek to assess the incremental cost effectiveness and long term impact of the conditional cash transfer element of the programme.

**Methods:** An impact analysis and incremental cost-effectiveness analysis of conditional cash transfers (CCTs) is undertaken taking a health service perspective toward costs of the intervention. The study was undertaken in Anambra state, comparing areas that received only the investment in health services with areas that implemented the conditional cash transfer programme. An interrupted time series analysis of the programme outputs was undertaken. These were combined with a programme costing to determine the incremental cost per output.

**Findings:** Maternal services provided to patients in conditional cash transfer areas accelerated rapidly from the middle of 2014 until after the programme in late 2015. The costs of providing services in each Primary Health Center facility was US $52,128 in the areas that only invested in health services compared to US $90,702 in facilities that also provided cash transfers. Much of the additional cost was in managing cash transfers. The incremental cost in the cash transfer areas was $572 for delivery care and $11 for antenatal care. If the programme was to be integrated as a regular service in the public health system, the cost of a delivery is estimated to fall to $389 and to $188 if 2015 levels of activity are assumed.

**Conclusion:** Although the cost of CCTs as originally constituted as a vertical programme are relatively high compared to other similar programmes, these would fall substantially if integrated into the main health system. There is also evidence of sustained impact beyond the end of the funding suggesting that short term programmes can lead to a long-term change in patterns of health seeking behavior.

## Introduction

The Subsidy Reinvestment and Empowerment Programme (SURE-P), Maternal and Child Health (MCH) (SURE-P MCH hereafter) was implemented in Nigeria from October 2012 to April 2015 in order to reduce neonatal and maternal mortality by increasing use of facility-based maternal and child health services. As well as support to service provision (supply-side), the programme provided a demand-side conditional cash transfer (CCT) to pregnant women as extra stimulus to utilize MCH services.

Demand-side health financing mechanisms, including vouchers for services and conditional cash transfers (CCT), are well-established tool for increasing use of health services (1, 2). Early CCTs in Latin America were provided to households on condition members accessed a range of services over an extended period of time providing a continued addition to household income (3).

Maternal CCTs are different in that they are restricted to payments before and just after childbirth. In this respect they are similar to most maternal voucher programmes which provide purchasing power, but not cash, for women to select facilities of their choice and often also provide other financial and non-financial benefits such as transport payments and items for the baby (4). Maternal vouchers in Bangladesh, India, Kenya and Uganda as well as the cash-transfer based Aama programme in Nepal provide similar benefits to the Nigeria SURE-P programme. There is good evidence that these schemes have generally increased utilization of skilled support for pregnancy and delivery (4, 5).

Sustainability of the demand-side health financing mechanisms remains a concern. Many have been dependent on funding from development partners, although in some of these countries, government has progressively taken over financing (5). Another issue is whether demand-side financing needs to be a permanent addition to the funding of health care or can it be withdrawn once individuals and communities have been accustomed to using services (6). The relatively short period of many evaluations means that the impact of withdrawing or downsizing a programme is often neglected.

We examine the impact, cost-effectiveness and longer term sustainability of the SURE-P MCH programme in Nigeria. This programme has now been discontinued and this feature allows us to look at the programme both during implementation and after it ceased to function. The paper has two objectives: (i) Understand the impact of the CCT programme on use of key maternal health services during the intervention and after its suspension compared with areas that only implemented the supply-side interventions; and (ii) understand the costs and incremental cost-effectiveness of the CCT programme compared to similar interventions elsewhere. The costing undertaken takes a health service perspective since a major focus is on how to sustain such programmes within the context of severely limited public health budgets.

In the next section we describe the programme and context of implementation. The following sections describe the data and methods used to analyse the programmes, results describing the costs and consequences of the programme in the selected areas and estimates of cost-effectiveness. Finally, the discussion seeks to compare the results to other similar programmes internationally and inform the future development of maternal policy.

SURE-P MCH was implemented in Nigeria between October 2012 and April 2015 (7). It was part of a package of social measures designed to cushion the removal of the national fuel subsidy. The programme was established by the Federal Government to improve access to quality maternal and child health services and respond to persistently high maternal and child mortality. SURE-P MCH was implemented in clusters in each state, comprised of 4 Primary Health Care (PHC) facilities linked to a referral hospital. SURE-P MCH comprises of both supply and demand side components. The supply side component focused on improvements to services at facilities including recruitment, training and deployment of skilled midwives, community health extension workers (CHEWs) and Village Health Workers (VHWs). It also provided investment in infrastructure development, improved availability of supplies and medicines and orientation of ward development committees (WDCs) to the needs of pregnant women and infants. The demand-side component aimed to stimulate use of maternal health services by giving cash to pregnant women who registered at a PHC center for antenatal check-ups, delivered at a public health facility, and took their baby for the first series of vaccinations. Theoretically, a woman could receive up to 5,000 Naira (USD 30), adjusted according to the number of services they used (7).

The implementation of the SURE-P MCH at the facility level started in October 2012 and CCTs were paid from March 2014. A new federal government ceased payments to states, including CCTs, in April 2015 although the programme was not officially stopped until November 2015. The present study was a component of a larger project, “Determinants of effectiveness and sustainability of a novel Community Health Workers programme in improving Mother and Child Health in Nigeria” (REVAMP), assessing the effectiveness and sustainability of the SURE-P MCH (8).

## Materials and Methods

The study was undertaken in Anambra state, southeast Nigeria, where SURE-P MCH was implemented in two clusters: one (SURE-P) cluster comprised of four PHC facilities (cluster 1: Nise, Okpuno, Umuawulu, and Uruogbo in Enugwu-Ukwu town) which received only the supply side interventions (SP) and a second cluster (CCT), also four facilities (cluster 2: Akwaeze, Awka-Etiti, Nnobi, and Oraeri), where the same supply side interventions was supplemented by conditional cash transfers (CCTs) to pregnant women. The clusters were selected, in consultation with district health officers (DHOs), to be similar to each other in terms of rurality and remoteness (Table 1). Detailed data collection was carried out from July 2016 to July 2018 with follow up collection of information from the Health Management Information System (HMIS) in March 2019 for the period after the programme had been closed.

**Table 1:** Description of the facilities.

	**Number of nurses and midwives**	**Number of CHEWS**	**Number of Village health workers**	**No of other staff**	**No of villages covered within facility catchment area**	**Location**
	**Cluster 1:SURE-P MCH only (SP)**					
Nise	5	4	6	3	3	Rural
Okpuno	5	3	6	1	4	Rural
Umuawulu	5	5	6	4	3	Rural
Uruogbo, Enugwu-Ukwu	5	3	6	0	2	Rural
	**Cluster 2:SURE-P MCH and CCT (CCT)**					
Akwaeze	5	3	6	2	6	Rural
Awka Etiti	6	5	7	1	4	Rural
Nnobi	5	3	6	7	7	Rural
Oraeri	7	2	6	1	5	Rural

*Rural is defined as a settlement with fewer than 20,000 inhabitants; other staff include cleaners and security personnel*.

*CHEWS, community health extension workers; MCH, maternal and child healthcare intervention; CCT, conditional cash transfer intervention*.

The study was a comparative retrospective study focusing on both the incremental costs and consequences (services provided) of the programme. The perspective of the study was on the additional costs to the health system and so does not incorporate the wider social costs of accessing services by the focal population. The programme aimed to increase the use of the main MCH services including antenatal and post-natal care, delivery with a skilled health worker and early childhood vaccination.

Information on these costs and consequences were obtained during facility visits from facility records. The consequences were tracked for 2 years beyond the end of the programme in order to understand the longer term effects on facility activity. The population in the areas of focus are largely rural. Antenatal care is almost universal, 99% according to a household survey conducted during the study (unpublished data from the REVAMP project). Delivery in public health facilities was around 57% with the remainder delivering with private providers or at home. A central objective of the SURE-P programme was to increase use of maternal services in public facilities, particularly amongst the poor.

A costing tool was developed to collect data on the demand and supply side costs of providing MCH services. The tool, piloted in Enugu State, had six sections: personnel; overheads; drugs and consumables; capital costs and CCT transfers. Data on costs were collected at primary health care, Local Government Area (LGA) and State levels in the two clusters for the year 2014 between November and December 2016. The incremental analysis considered only those costs that were additional to those all already spent on health services in the area.

Staff time was apportioned to different services by asking midwives, CHEWS and health assistants about working patterns and examining their shift schedules. The total numbers of working hours were disaggregated into key MCH service deliveries: antenatal care (ANC); delivery; post-natal care (PNC); and other activities. The working week was found to be divided into regular clinics for antenatal care, immunization and family planning. The time spent by members of staff in each clinic enabled an allocation of time to these activities. Staff were questioned about the amount of time spent on deliveries during a week. The remainder of the working time of each member of staff was allocated to general (non-MCH) services.

Conditional Cash Transfers were paid to women throughout 2014 and for three and a half months of 2015. The cost of CCTs comprised the actual CCT that was paid out to women and the costs of supporting the administration of the CCT programme. Administrative support included: (i) administrative personnel cost including salaries of the supervisor, field officer and technical officers at the state level; (ii) logistics costs including CCT registers, folders and cards, tally sheet and other consumables; (iii) training of staff involved in the administering the programme (treated as a capital cost). The cost of training, redeployment and salaries of the state level CCT staff was shared across the 4 CCT facilities, while the cost of SURE-P program officer was apportioned to 12 health centers where the intervention was carried out and the cost reflected in the 4 SURE-P MCH and CCT clusters.

Capital costs at the facilities were categorized into building renovation, vehicle, medical equipment, and training of staff on SURE-P activities. A list of medical equipment, infrastructure and supplies that were provided to each facility during SURE-P period was collected from each of the facilities. Building renovation costs were annualized over a 30-year period and equipment over 5 years. A 6 percent base discount rate is used in annualization, which equates to the real return on Nigerian government treasury bonds (14% return minus 8% inflation) in 2014.

A price list for drugs and consumables that were supplied by SURE-P MCH were collected from each facility. Drugs and consumables were also supplied to these facilities by the State Ministry of Health through the local government. Some items, particularly equipment and some supplies, were provided to facilities in-kind. In these cases, their value was imputed from a market survey and prices held at the Central Medical Store (CMS) Enugu for the year 2014 (see Table A1). Total and incremental costs are expressed in US dollars based on Oanda currency convertor of the Naira to dollar rate in June 2014 (www1.oanda.com/currency/converter/).

Information on consequences was obtained from routine Health Management Information System (HMIS) data in each of the facilities. HMIS data contained information on each of the key MCH components—antenatal, delivery and post-natal care. Post-natal care included both the checks made on infants soon after birth and vaccinations provided during the first year of life.

Monthly HMIS data were aggregated by cluster type to provide annual before (2012) and after (2014) consequences for the economic evaluation. We constructed monthly, cluster-level time-series for the main outcomes to examine whether these outcomes were associated with the introduction and withdrawal of SURE-P. Each monthly, cluster-level time-series outcome was computed by summing the facility-level outcome values (e.g., the total number of facility deliveries) each month that the time series data were available for all facilities within each cluster. An interrupted time series (ITS) analysis was used to analyse these outcomes by allowing for immediate and gradual (trend) build effects. The CCT cluster (CCT) was compared with the cluster (SP) that only implemented the SURE-P MCH supply-side intervention. The time series runs from October 2012 when the SURE-P MCH programme began until end of 2017, more than 2 years after SURE-P funding was discontinued. Policy interruptions are tested at the point CCTs began to be paid in March 2014 (*XS*) and the removal of funding in April 2015 (*XF*). The ITS equation is specified as:

$$\begin{array}{l} O_{t}=\;\beta_{o}+\;\beta_{1}t+\;\beta_{2}Z+\;\beta_{3}Z\;t\;+\;\beta_{4}XS_{t}+\;\beta_{5}\;XS_{t}t\\ \;+\;\beta_{6}XS_{t}Z+\;\beta_{7}\;XS_{t}\;Z\;t+\beta_{8\;}XF_{t}+\;\beta_{9}\;XF_{t}t\\ \;+\;\beta_{10}XF_{t}Z+\;\beta_{11}\;XF_{t\;}Z\;t+\overset{12}{\underset{i=2}{\sum }}\gamma_{i}m_{i}\;+\;\varepsilon_{t} \end{array}$$

Where *t* is a time trend *Z* is a dummy variable to indicate an intervention (1 = CCT) or comparison area (0 = SP), *XS* is a dummy to indicate a time period after the CCT intervention was implemented (0 = before, 1 = after), *XF* is a dummy to indicate a time period after the withdrawal of the CCT intervention (0 = before, 1 = after), m2–m12 (*m*_*i*_) are monthly dummies to take account of the potentially seasonal nature of the data and ε_*t*_ is an error term.

The impact of the introduction of CCT is indicated by an immediate effect interaction term *XS*_*t*_ *Z* and a gradual (slope) effect interaction term *XS*_*t*_ *Z t*, while the effect of the removal of CCT is measured by an immediate effect interaction term *XF*_*t*_*Z* and a gradual (slope) effect interaction term *XF*_*t*_ *Z t*. Therefore, these interaction terms are difference in differences (DiD) estimates. More specifically, for immediate changes in level they estimate the difference between: (a) the difference in the immediate level (mean value) of the outcome in the CCT cluster at the start of the period following the interruption compared to the start of the period preceding the interruption, and (b) the difference in the immediate level (mean value) of the outcome in the SP cluster at the start of the period following the interruption compared to the start of the period preceding the interruption. While for changes in trend they estimate the difference between: (a) the difference in the mean linear trend of the outcome in the CCT cluster during the period following the interruption compared to the period preceding the interruption, and (b) the difference in the mean linear trend of the outcome in the SP cluster during the period following the interruption compared to the period preceding the interruption.

The model and all its coefficients (see ITS equation above) was estimated as an ordinary least squares regression using the *itsa* function in Stata (9). The p-values for the key model coefficients were estimated based on the *t*-statistic. The specification is tested for generalized serial correlation (using the *actest* in Stata) and adjustments were made to the lag structure if necessary (10). The model is estimated for the period from October 2012 (when SURE-P was introduced) to December 2017.

Average and incremental costs of maternal services are calculated. The average cost is based on the total cost of services apportioned to each activity divided by the units of output. The incremental cost is based on the additional cost incurred in the CCT areas for SURE-P services ($C_{1}^{cct}-\;C_{0}^{cct}$) less the cost in SURE-P areas without cash transfers ($C_{1}^{sp}-\;C_{0}^{sp}$). Incremental activity is based on a difference-in-difference calculation comparing the activity in the year before and after intervention in CCT areas ($A_{1}^{cct}-\;A_{0}^{cct}$) with the same difference for SURE-P only areas ($A_{1}^{sp}-\;A_{0}^{sp}$). Incremental cost effectiveness is calculated as:

$$\begin{array}{l} ICER=\frac{\left(C_{1}^{cct}-\;C_{0}^{cct}\right)-\left(C_{1}^{sp}-\;C_{0}^{sp}\right)}{\left(A_{1}^{cct}-\;A_{0}^{cct}\right)-\left(A_{1}^{sp}-\;A_{0}^{sp}\;\right)} \end{array}$$

Two scenarios for incremental costs of CCTs are calculated based on the number of transfers given out during the first year of the programme: (i) administering the CCT mechanism as a vertical programme including the cash transfers themselves, additional personnel cost used to administer the programme and consumable and small capital costs needed to manage the programme; (ii) administering the CCT mechanism as part of the routine activities of the health facility so that parallel staffing are not required. A third scenario is also calculate based on the higher number of CCTs allocated during the first part of 2015. Sensitivity analysis around the base discount rate of 6% was undertaken with the rate varied between 2 and 10%.

Facility level data on the supply-side is supplemented by results from a community survey of women that gave birth during and just after the SURE-P programme. The survey was based on a community listing of all households in the project cluster areas that had a birth in the last 6 years; covering a period before, during and after the SURE-P programme. A stratified random sample of 713 women were selected for interview across the three project areas. The questionnaire, which collected information on maternal health seeking behavior, attitudes to the care given and socioeconomic information on the household was administered in May 2018. All tools listed are part of a larger realist evaluation of the SURE-P programme (8). Ethics approval for the study was obtained from the University of Leeds School of Medicine and University of Nigeria ethics committees.

## Results

**Figure 1:**
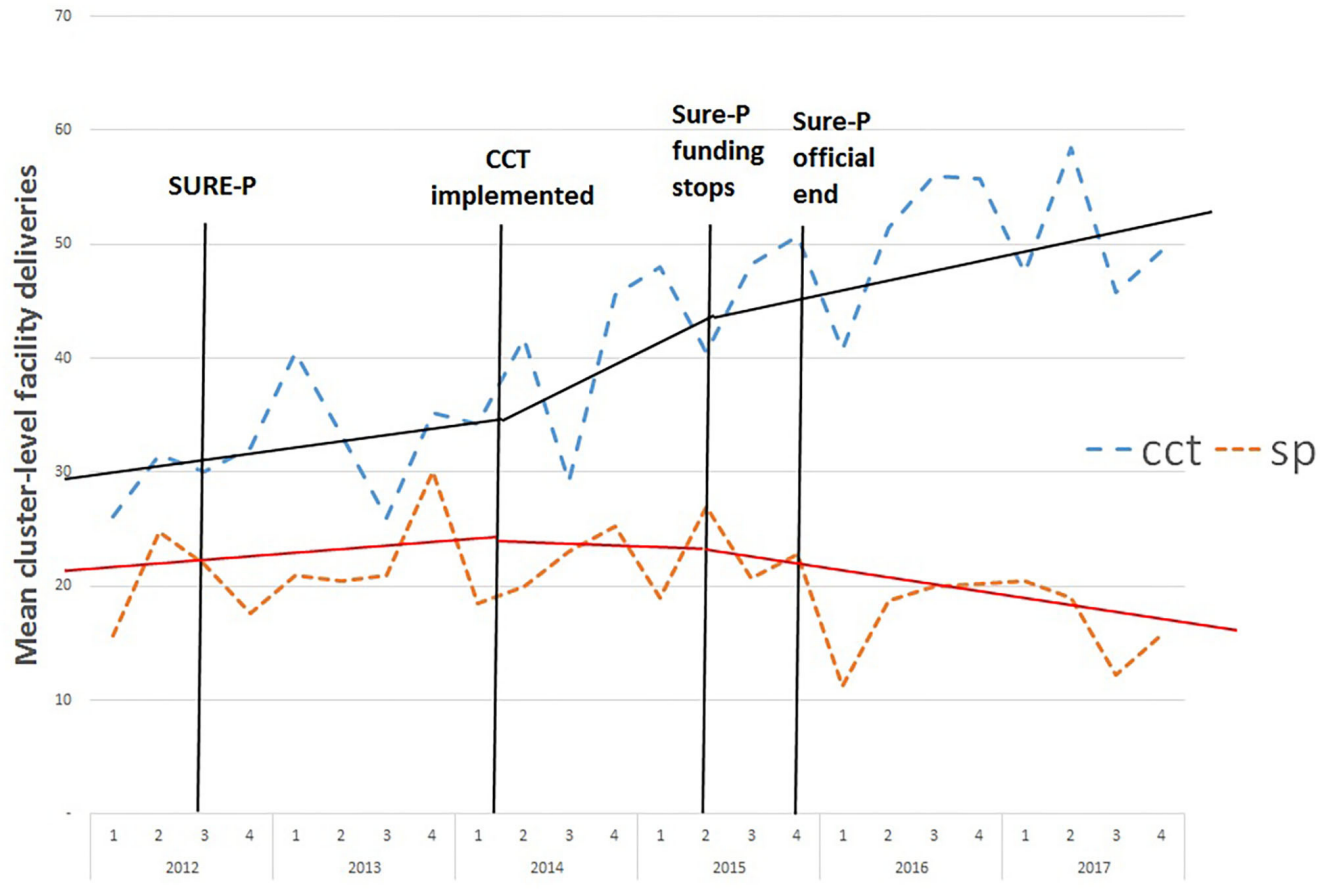
Monthly average deliveries in each facility in CCT and SP clusters before, during and after the SURE-P MCH programme. SP, SURE-P MCH cluster, i.e., monthly outcomes from facilities that only implemented the SURE-P MCH (maternal and child healthcare) intervention alone; CCT, SURE-P MCH and CCT cluster, i.e., monthly outcomes from facilities that implemented both the SURE-P MCH (maternal and child healthcare) and CCT (conditional cash transfer) interventions; SURE-P, Subsidy Reinvestment and Empowerment Programme. On the x-axis the numbers 1–4 above the years indicate the quarters within each year, i.e., the four three-monthly periods within each year from the start of the year to the end. The dashed trend lines (blue/orange) indicate the trend in the raw/exact values of the monthly, cluster-level outcome in the CCT/SP clusters. The solid trend lines (black/red) indicate the linear trend estimated by the ITS-model within the periods before, during and after the CCT intervention was implemented within the CCT cluster, and the model-estimated change in those trends (including any immediate change in level and any change in slope) between each of these periods, for the CCT/SP clusters.

Examination of the graphical trend in facility deliveries suggest that prior to the start of the CCT programme, similar trends activity can be observed in both the CCT and SP groups. Following introduction of CCTs, there appears to be a sharp increase in deliveries in the CCT areas while no such trend is observed in SP areas (Figure 1).

**Figure 2:**
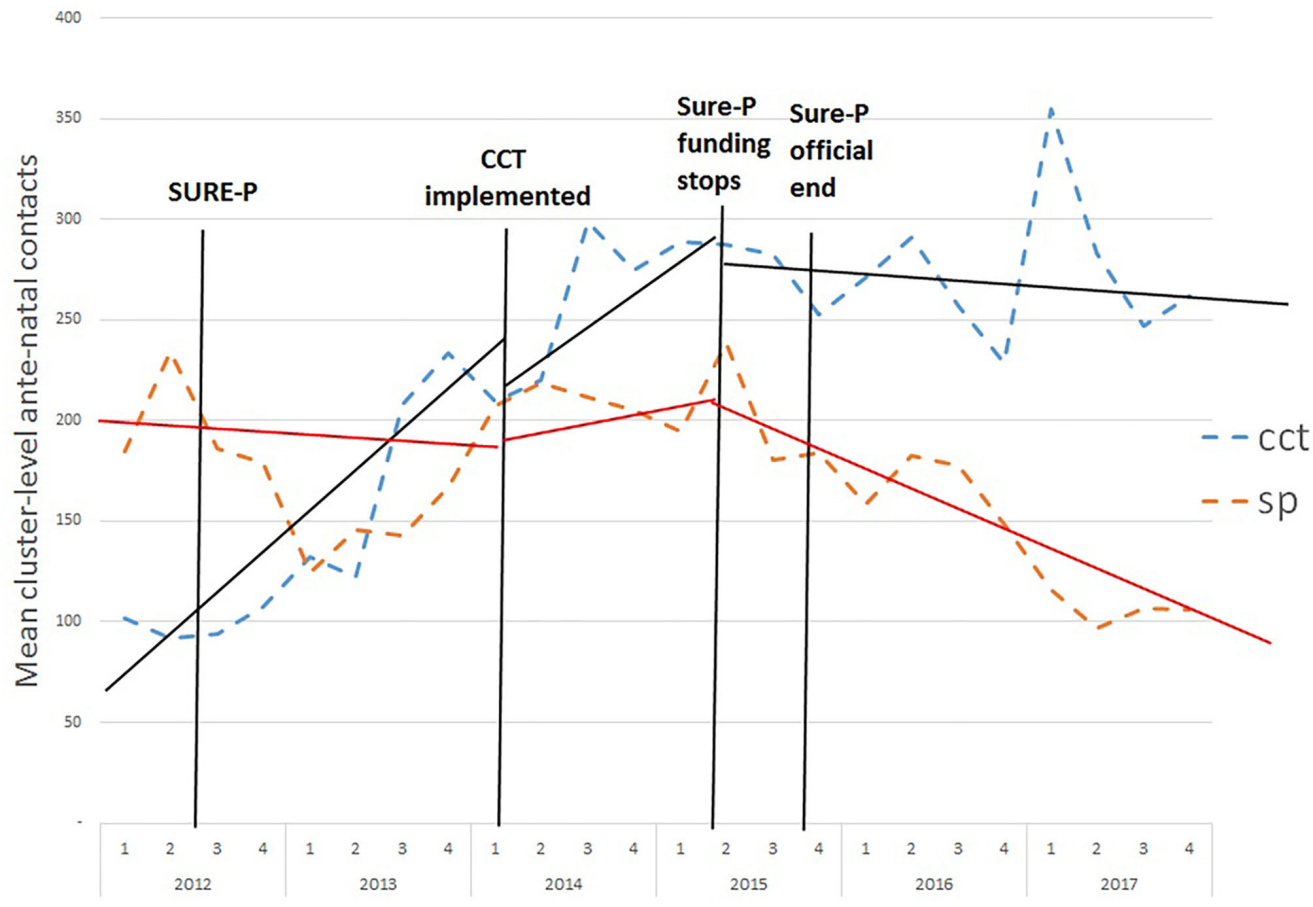
Monthly average antenatal contacts in each facility in CCT and SP clusters before, during and after the SURE-P MCH programme. SP, SURE-P MCH cluster, i.e., monthly outcomes from facilities that only implemented the SURE-P MCH (maternal and child healthcare) intervention alone; CCT, SURE-P MCH and CCT cluster, i.e., monthly outcomes from facilities that implemented both the SURE-P MCH (maternal and child healthcare) and CCT (conditional cash transfer) interventions; SURE-P, Subsidy Reinvestment and Empowerment Programme. On the x-axis the numbers 1–4 above the years indicate the quarters within each year, i.e., the four three-monthly periods within each year from the start of the year to the end. The dashed trend lines (blue/orange) indicate the trend in the raw/exact values of the monthly, cluster-level outcome in the CCT/SP clusters. The solid trend lines (black/red) indicate the linear trend estimated by the ITS-model within the periods before, during and after the CCT intervention was implemented within the CCT cluster, and the model-estimated change in those trends (including any immediate change in level and any change in slope) between each of these periods, for the CCT/SP clusters.

For antenatal visits, the picture is less clear cut with visits in CCT areas rising sharply well before the introduction of the CCTs (Figure 2). There is also a leveling off or decline after the end of the programme although the downward trend is larger in the SP areas. The weaker or absent association with the CCT intervention is perhaps unsurprising since access to CCTs was made available during ANC care so that, particularly in the early stages of policy, it is likely that women may be less aware of the policy until their first ANC visit.

**Figure 3:**
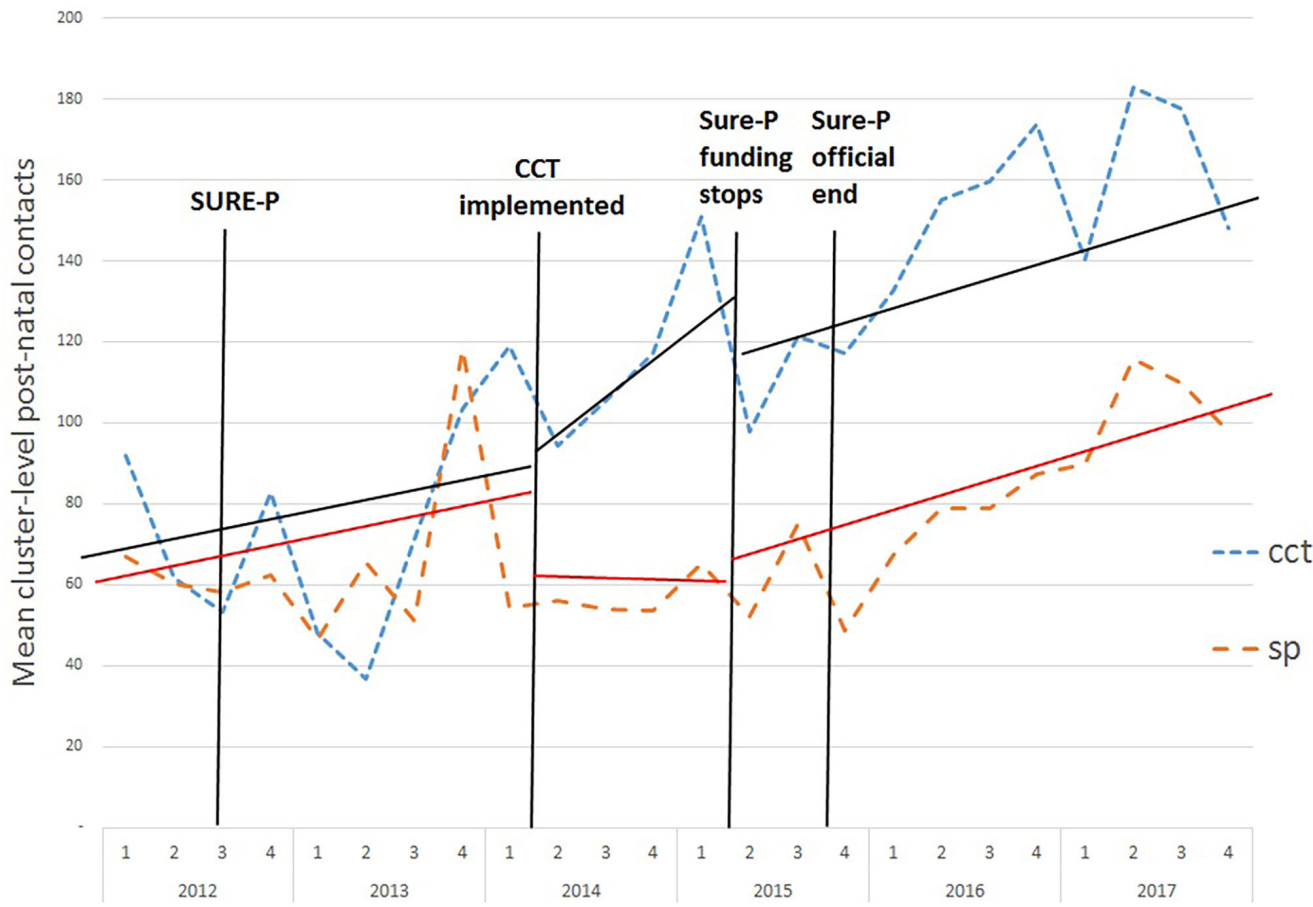
Monthly average post-natal contacts in CCT and SP clusters before, during and after the SURE-P MCH programme. SP, SURE-P MCH cluster, i.e., monthly outcomes from facilities that only implemented the SURE-P MCH (maternal and child healthcare) intervention alone; CCT, SURE-P MCH and CCT cluster, i.e., monthly outcomes from facilities that implemented both the SURE-P MCH (maternal and child healthcare) and CCT (conditional cash transfer) interventions; SURE-P, Subsidy Reinvestment and Empowerment Programme. On the x-axis the numbers 1–4 above the years indicate the quarters within each year, i.e., the four three-monthly periods within each year from the start of the year to the end. The dashed trend lines (blue/orange) indicate the trend in the raw/exact values of the monthly, cluster-level outcome in the CCT/SP clusters. The solid trend lines (black/red) indicate the linear trend estimated by the ITS-model within the periods before, during and after the CCT intervention was implemented within the CCT cluster, and the model-estimated change in those trends (including any immediate change in level and any change in slope) between each of these periods, for the CCT/SP clusters.

The number of PNC contacts appears to increase substantially in CCT areas soon after the SURE-P policy was introduced (Figure 3).

These visual conclusions are reinforced by the ITS calculations (Table 2). The ITS is estimated with two interruptions: at the introduction of the cash transfers and at their suspension. The Cumby-Huizinga test (available as the *actest* function in Stata) for autocorrelation suggests that adjusting for a one period lag is sufficient to eliminate the serial correlation.

**Table 2:** Interrupted Time Series results—coefficients and comparison of linear post-intervention trends.

		**Facility deliveries**			**ANC contacts**			**PNC contacts**		
**Variable description**	**Variable**	**Coef**	***t***		**Coef**	***t***		**Coef**	***t***	
General time trend	t	0.22	4.79	***	0.03	0.06		1.14	3.61	***
Dummy intercept for CCT area	Z	5.93	7.32	***	−21.12	−3.97	***	2.16	0.5	
General time trend for CCT area	Z t	−0.29	−2.91	***	3.10	4.88	***	−0.30	−0.53	
Dummy intercept for post-implementation period	XS	−2.52	−3.15	***	21.57	3.32	***	−14.26	−4.24	***
General time trend for the implementation period	XS t	−0.16	−2.28	**	−0.46	−0.96		−0.95	−2.25	**
DiD immediate change: CCT introduced	X S Z	2.94	2.13	**	−31.89	−4.19	***	18.32	2.91	***
DiD trend change: CCT introduced	XS Z t	0.57	3.51	***	−0.19	−0.3		0.95	0.95	
Dummy intercept for withdrawal period	XF	0.00	0		7.11	1.76	*	−3.07	−1.15	
General time for the withdrawal period	XF t	−0.15	−2.56	**	−0.93	−3.14	***	0.44	1.96	*
DiD immediate change: CCT withdrawn	XF Z	−0.83	−0.47		−20.90	−3.89	***	−5.39	−0.74	
DiD trend change: CCT withdrawn	XF Z t	−0.12	−1.09		−1.60	−3.435	***	−0.57	−0.9	
Dummies for m2–m12 included but not reported										

Significant at the ***1% level, **5% level, *10% level.

*ANC, antenatal care; PNC, post-natal care; Coef, linear regression model coefficient for each covariate; t, t-statistic value associated with each covariate's coefficient; CCT, SURE-P MCH and CCT cluster, i.e., cluster facilities included SURE-P MCH (maternal and child healthcare) and CCT (conditional cash transfer) interventions; DiD, difference-in-differences; m2–m12, month 2–month 12*.

The ITS suggests there was a significant increase (2.94, *p* < 0.05) in the mean monthly number of deliveries in the CCT cluster when the CCT intervention began compared to the level at the start of the time series (after controlling for the equivalent comparison in the SP cluster). Similarly, there was a significant but small increase (*p* < 0.01, 0.57) in the monthly trend in the number of deliveries in the CCT cluster during the period when the CCT intervention was implemented compared to before it was introduced (after controlling for the equivalent comparison in the SP cluster).

The ITS suggests no clear evidence for any change in the monthly number of facility deliveries immediately following the termination of the CCT intervention compared to the level when CCTs were first implemented (after controlling for the equivalent comparison in the SP cluster). There was also no evidence for any clear change in the trend in the monthly number of facility deliveries during the period following the termination of the programme. This suggests that the effects of the intervention may be relatively resilient despite finance for the system coming to an end.

For ANC care, there is an upward trend in CCT areas throughout the time series which is largely independent of the introduction and later abolition of the CCT policy. The ITS suggests a statistically significant (−31.9, *p* < 0.01) immediate reduction in visits after the implementation of CCTs.

For PNC contacts the upward trend appears to affect both areas although there is an initial increase (18.3, *p* < 0.01) immediately after the introduction of cash transfers in CCT areas. The DiD effects when the programme ended are not statistically significant.

The SURE-P programme provided substantial additional resources for public services: increasing resources in SURE-P/only areas by 152% and CCT areas by 309% (Table 3). Much of the additional resource was to fund staff positions including Midwives, CHEWS, Village health workers salaries and trainings; drugs and consumables; capital projects and in CCT areas to pay for the cash transfers and their administration.

**Table 3:** Total costs of services in SP and CCT facilities (US Dollars per facility).

	**Pre sure/P**		**SP facilities**		**CCT facilities**	
	**$**	**%**	**$**	**%**	**$**	**%**
**Costs by line item**						
Personnel	$ 12,462	56%	$ 33,823	60%	$ 36,841	41%
Capital	$ 7,691	35%	$ 11,153	20%	$ 14,814	16%
Drugs and consumables	$ 2,128	10%	$ 11,153	20%	$ 25,602	28%
CCT activities					$ 13,446	15%
Total Cost	$ 22,282	100%	$ 56,128	100%	$ 90,702	100%
**Costs by activities**						
ANC	$ 4,150	19%	$ 10,656	19%	$ 18,493	20%
Delivery	$ 6,609	30%	$ 14,164	25%	$ 30,868	34%
PNC	$ 4,472	20%	$ 9,755	17%	$ 20,012	22%
Other activities	$ 7,050	32%	$ 21,554	38%	$ 21,330	24%
Total cost	$ 22,282	100%	$ 56,128	100%	$ 90,702	100%
Percentage change in total cost			152%		307%	

*SP, SURE-P MCH cluster, i.e., cluster facilities included only SURE-P MCH (maternal and child healthcare) intervention; CCT, SURE-P MCH and CCT cluster, i.e., cluster facilities included SURE-P MCH (maternal and child healthcare) and CCT (conditional cash transfer) interventions; ANC, antenatal care; PNC, post-natal care*.

The costs of providing services in each PHC facility was US $56,128 in the SURE-P only areas compared to US $90,702 in facilities that also provided CCTs (Table 3). Much of the additional cost in CCT facilities was the costs of providing CCTs although spending on drugs and consumables was also notably higher. The study found that in SP facilities around 62% of the costs can be attributed to maternal care (ANC, delivery and PNC) whilst in CCT facilities this proportion increases to 76%.

The CCT programme was administered as a vertical programme with its own staff and training requirements. Over the course of CCT implementation, the cost of the cash transfers to beneficiaries themselves amounted to only around 20% of the total cost of the programme (Table 4). The main cost (74%) was in employing staff to manage the programme, targeting beneficiaries and making payments.

**Table 4:** The cost components for implementing CCTs (2014–2015 quarter 1, US $).

**Health centers**	**Cash transfers**	**CCT personnel cost**	**Logistics**	**Total**
Akwaeze	3,094	12,872	1,059	17,024
Awka Etiti	5,594	12,872	1,090	19,556
Nnobi	3,763	12,872	1,115	17,749
Oraeri	1,219	12,872	1,053	15,143
Total	13,669	51,488	4,316	69,473
% of total	20%	74%	6%	100%

*CCT, Conditional Cash Transfer*.

Table 5 shows that the average cost of a delivery in 2014 was $205 (Naira 32,762) in CCT facilities compared to $163 (N 26,123) in SP facilities while for ANC visits the respective cost was in $18 (N 2,951) and $13 (N2,022). A smaller number of PNC visits in CCT facilities mean that the cost of PNC was more than double the cost in SURE-P/only facilities. If a broader definition of PNC is used (PNC plus) that includes immunizations and other services for the new-born the cost differential is substantially lower.

**Table 5:** Average and incremental costs of ANC and delivery.

	**Average costs**		**Incremental costs of CCTs**	
	**SP**	**CCT**	**Vertical programme (2014 activity)**	**Integrated programme (2014 activity)**
**Naira**				
ANC	2,022	2,951	1,838	1,068
Delivery	26,123	32,762	91,550	62,218
PNC	15,190	31,545	NA	NA
PNC plus	2,446	3,135	NA	NA
**US dollars**				
ANC	$ 13	$ 18	$ 11	$ 7
Delivery	$ 163	$ 205	$ 572	$ 389
PNC	$ 95	$ 197	NA	NA
PNC plus	$ 15	$ 20	NA	NA

*An incremental unit cost for PNC could not be computed since data from before the programme is not complete*.

*CCT, SURE-P MCH and CCT cluster, i.e., cluster facilities included SURE-P MCH (maternal and child healthcare) and CCT (conditional cash transfer) interventions; SP, SURE-P MCH cluster, i.e., cluster facilities included only SURE-P MCH (maternal and child healthcare) intervention; ANC, antenatal care; PNC, post-natal care; PNC plus, post-natal care plus immunizations and other services for the new-born*.

Managing the CCT mechanism as a vertical programme and including the consequent costs implies an incremental cost of $572 (N 91,550) for delivery care and $11 (N 1,838) for ANC care. These costs are inflated by the administration required to run a vertical programme. If it is assumed that the programme can be administered within the existing system, so the main additional costs are restricted to cash transfers and administrative logistics, the incremental cost falls to $389 (N 62,218). Activity in CCT areas in particular accelerated quite rapidly from the middle of 2014 until after the programme was abandoned in late 2015. If the activity for the latter quarter of 2014 to middle of 2015 is used as the denominator, then after adjusting the 2014 costs for the higher levels of activity in 2015 the incremental cost of delivery falls to $188 (N 30,1447).

Sensitivity around the discount rate made little difference to the ICER. Varying the discount rate from 2 to 10% changes the incremental cost per ANC (assuming it is implemented as a vertical programme) from $11.3 to $11.7 while the incremental cost per delivery varies from $566 to $578.

## Discussion

Data both from our clusters and national assessments suggest that introduction of SURE-P was associated with an increased use of public facilities for deliveries in CCT areas but not in areas only receiving supply-side investment. An early evaluation, ending in March 2014, could only find statistically significant increases (compared to comparator clusters) for antenatal care and vaccinations (11). Our data for the CCT clusters in Anambra state suggest a statistically significant increase in both facility deliveries and antenatal care over the entire programme period (October 2014 to March 2015) when compared to other SURE-P areas. A CCT effect, around the time funding for the policy began, is found for facility deliveries but not ANC. The ITS suggests that the increased deliveries coincided with the introduction of cash transfers but induced a small gradual increase rather than dramatic change in utilization.

Our study suggests that the cost for every additional delivery under the CCT programme was $572 as part of a vertical programme but might fall if integrated into the health system. This cost appears high when compared to other similar programmes. In Bangladesh, for example, an evaluation of a maternal voucher scheme found an ICER of $70 per delivery (12). An assessment of a voucher scheme in India found a cost of Rs. 3,533 ($63) per delivery although it is unclear whether this represents an average or incremental cost (13).

Two important qualifications to the findings on incremental cost are required. Firstly, the programme was ended for political reasons early in its life. Much of the cost of the transfer programme can be considered fixed. There are signs that in the quarters before the end of the programme the incremental cost would fall substantially. Such reductions are in line with other similar programmes. In the early Progresa scheme in Mexico, for example, the administrative costs in the first year exceeded 130% of the benefits to beneficiaries but fell to <5% after a few years (14). We calculate that over the first year, the administrative cost of the SURE-P CCT programme represented 370% of the value of CCTs distributed. If these costs are absorbed into the main health system, then the ICER per delivery for the 2014 would fall to around $389 or $188 if the higher activity levels in 2015 are assumed.

A second issue concerns the extent to which the large up-front cost can be considered not only as a way of encouraging current use of skilled delivery services but as an investment that generates a longer term impact on health seeking behavior going well-beyond the end of the programme. The timing of the study allows us to examine what happened after the ending of funding for the intervention. Information on use of services 2 years beyond the end of the programme suggest that the effect of the intervention may have continued well-beyond the period of funding. The investment in the supply side into equipment and the facility was not immediately lost when the programme closed so potentially explaining the continued effect. It is notable, however, that the persistent effect is largely evident in the CCT areas and not the SP areas which also received the supply side investment. This finding is supported by a community survey undertaken as part of the wider realistic evaluation of the SURE-P programme: 70% of women living in CCT areas said that their trust in public services increased as a result of the introduction of CCTs compared to 49% in SURE-P only areas (15). Following withdrawal of CCTs, the majority of those women in those areas (62%) reported continued high levels of trust. This is reinforced by patterns of delivery which suggested that there was no statistical difference in the proportion of women reporting using a public facility during and after the CCT programme (69 ± 6.4% before compared to 64 ± 7.8% after).

The experience of the areas post-SURE-P raises a wider question about the extent to which the demand-side initiatives become a permanent part of the system or should be considered a short-term mechanism for promoting use of services. Studies have shown that it depends on what the critical barriers to utilization are. It is therefore necessary to understand these, in order to devise effective solutions (6). There is also the need to build in evaluation mechanisms while designing such programs, to provide evidence-based tools for advocacy for sustainability of programs found to be effective (3).

This study has a number of limitations. The detailed costing data collected was limited to 1 year (2014) although inspection of budgets suggest that apart from SURE-P most spending follows a historical pattern with small increments each year for inflation. The results presented are based on information from a case study in two areas of Anambra State and are not necessarily representative of all the areas where SURE-P was implemented. The study assessed the quantity of services used, the place of service delivery and perceptions of the community about the quality and responsiveness of services. It did not attempt to assess quality of care in a technical sense either before or after the policy implementation. The lack of quality assessment in many studies of demand-side financing, suggests that this represents one area for future research in the Nigerian and other contexts.

## Conclusion

This study has provided new information on the costs and consequences of using a comprehensive intervention to improve provision and access to MCH services through the public PHC centers and the incremental cost and outputs from the SURE-P MCH programme. It is one of the few studies to assess a programme for an extended period after funding and formal implementation ceased. Although the cost of CCTs as originally constituted as a vertical programme are relatively high, the review of the cost structure suggests that these would fall substantially if it had been integrated into the main health system. Furthermore, there is evidence that the effects of CCTs persist beyond the initial impact on direct beneficiaries with a general increase in the willingness to use health facilities for maternal care. Such effects may be associated with a general build up in trust in the system.

## Data Availability Statement

The raw data supporting the conclusions of this article will be made available by the authors, without undue reservation.

## Ethics Statement

The studies involving human participants were reviewed and approved by School of Medicine, University of Leeds and Health Research Ethics Committee, University of Nigeria Teaching Hospital. The patients/participants provided their written informed consent to participate in this study.

## Author Contributions

OO, JH, and TE conceived and oversaw the analysis and drafting of the manuscript. PO, UE, CO, BE, TM, BU, and EE helped develop the data collection instruments, collected data, and helped analyse the costs. All authors read and approved the final version of the manuscript.

## Conflict of Interest

The authors declare that the research was conducted in the absence of any commercial or financial relationships that could be construed as a potential conflict of interest.

## Appendix

**Table A1:** Methods for deriving costs of all the inputs.

**Costs data**	**Recurrent**
Personnel	Monthly and annual staff salaries including their grade levels were retrieved from facility health workers and were cross checked from the state and LGA salary structure of each health worker for the year 2104. The share of the personnel costs for MCH (ANC, PNC, delivery and other activities) services were determined by allocating time (in hours) to each of the MCH component and working it out with annual staff salaries.
Drugs and consumables	A uniform price list for MCH drugs and consumables used was sourced from the CMS/Enugu State's drug revolving fund systems for 2014.
Overhead	Monthly and annual expenditure on administration and overheads including transport & communication, utility, printing & stationery, maintenance, fuel & lubricant, etc. Unit costs were determined by 2014 market price.
CCT Activities	The demand side costs were those borne by SURE-P in carrying out their activities in the facilities. Some of these costs include those of state level recruitment of staff, the amount spent on training these state level personnel, redeployment information for each of the facilities, cost of program administration, and cost of paying beneficiaries of CCT (Facility Level).
**Cost data**	**Capital**
Building	The costs of new buildings were determined using the price of a standard PHC building (reference) and by estimation.
Renovations	The costs of renovating old building made by SURE P MCH programme in all SURE P MCH clusters were obtained from the facility records. Such renovations included: window and door repairs, roofing and ceiling, tiling, painting etc.
Medical Equipment	2014 market price was used to obtain cost of medical equipment available in each facility under study
Motor vehicle	Unit cost of motor vehicles (bicycles and motor cycles) were obtained using 2014 market price list from market survey.
Generator	Unit cost of generator was obtained using 2014 market price list obtained from market survey.
Borehole	The cost of borehole project was obtained by the price of digging a standard borehole in 2014 in facilities where boreholes were dug.
Others	Unit cost of other capital items such as furniture, water storage tanks and stand, solar energy, refrigerators and deep freezers etc. was obtained using 2014 market price list obtained from market survey.

*LGA, local government area; ANC, antenatal care; PNC, post-natal care; MCH, maternal and child healthcare; SURE-P, Subsidy Reinvestment and Empowerment Programme; CCT, SURE-P MCH and CCT cluster, i.e., cluster facilities included SURE-P MCH (maternal and child health) and CCT (conditional cash transfer) interventions*.
